# Validation of the North Star Assessment for Limb-Girdle Type Muscular Dystrophies

**DOI:** 10.1093/ptj/pzac113

**Published:** 2022-08-06

**Authors:** Meredith K James, Lindsay N Alfano, Robert Muni-Lofra, Natalie F Reash, Jassi Sodhi, Megan A Iammarino, Dionne Moat, Kianna Shannon, Michelle McCallum, Mark Richardson, Michelle Eagle, Volker Straub, Chiara Marini-Bettolo, Linda P Lowes, Anna G Mayhew

**Affiliations:** The John Walton Muscular Dystrophy Research Centre, Newcastle University and Newcastle Hospitals NHS Foundation Trust, Newcastle upon Tyne, UK; Center for Gene Therapy, The Abigail Wexner Research Institute at Nationwide Children’s Hospital, Columbus, Ohio, USA; Department of Pediatrics, The Ohio State University, Columbus, Ohio, USA; The John Walton Muscular Dystrophy Research Centre, Newcastle University and Newcastle Hospitals NHS Foundation Trust, Newcastle upon Tyne, UK; Center for Gene Therapy, The Abigail Wexner Research Institute at Nationwide Children’s Hospital, Columbus, Ohio, USA; The John Walton Muscular Dystrophy Research Centre, Newcastle University and Newcastle Hospitals NHS Foundation Trust, Newcastle upon Tyne, UK; Center for Gene Therapy, The Abigail Wexner Research Institute at Nationwide Children’s Hospital, Columbus, Ohio, USA; The John Walton Muscular Dystrophy Research Centre, Newcastle University and Newcastle Hospitals NHS Foundation Trust, Newcastle upon Tyne, UK; Center for Gene Therapy, The Abigail Wexner Research Institute at Nationwide Children’s Hospital, Columbus, Ohio, USA; The John Walton Muscular Dystrophy Research Centre, Newcastle University and Newcastle Hospitals NHS Foundation Trust, Newcastle upon Tyne, UK; The John Walton Muscular Dystrophy Research Centre, Newcastle University and Newcastle Hospitals NHS Foundation Trust, Newcastle upon Tyne, UK; The John Walton Muscular Dystrophy Research Centre, Newcastle University and Newcastle Hospitals NHS Foundation Trust, Newcastle upon Tyne, UK; The John Walton Muscular Dystrophy Research Centre, Newcastle University and Newcastle Hospitals NHS Foundation Trust, Newcastle upon Tyne, UK; The John Walton Muscular Dystrophy Research Centre, Newcastle University and Newcastle Hospitals NHS Foundation Trust, Newcastle upon Tyne, UK; Center for Gene Therapy, The Abigail Wexner Research Institute at Nationwide Children’s Hospital, Columbus, Ohio, USA; Department of Pediatrics, The Ohio State University, Columbus, Ohio, USA; The John Walton Muscular Dystrophy Research Centre, Newcastle University and Newcastle Hospitals NHS Foundation Trust, Newcastle upon Tyne, UK

**Keywords:** Clinician-Reported Outcome Measure, Limb-Girdle Muscular Dystrophy, Motor Performance, Neuromuscular Diseases, Rasch Methods

## Abstract

**Objective:**

The North Star Assessment for limb-girdle type muscular dystrophies (NSAD), a clinician-reported outcome measure (ClinRO) of motor performance, was initially developed and validated for use in dysferlinopathy, an autosomal recessive form of limb-girdle muscular dystrophy (LGMD R2/2B). Recent developments in treatments for limb-girdle muscular dystrophies (LGMD) have highlighted the urgent need for disease-specific ClinROs. The purpose of this study was to understand the ability of the NSAD to quantify motor function across the broad spectrum of LGMD phenotypes.

**Methods:**

Assessments of 130 individuals with LGMD evaluated by the physical therapy teams at Nationwide Children’s Hospital and the John Walton Muscular Dystrophy Research Centre were included in the analysis. NSAD, 100-m timed test (100MTT), and Performance of Upper Limb 2.0 assessment data were collected. Psychometric analysis with Rasch measurement methods was used to examine the NSAD for suitability and robustness by determining the extent to which the observed data “fit” with predictions of those ratings from the Rasch model. The NSAD score was correlated with the 100MTT and Performance of Upper Limb 2.0 assessment scores for external construct validity.

**Results:**

The NSAD demonstrated a good spread of items covering a continuum of abilities across both individuals who had LGMD and were ambulatory and individuals who had LGMD and were weaker and nonambulatory. Items fit well with the construct measured, validating a summed total score. The NSAD had excellent interrater reliability [intraclass correlation coefficient (ICC) = 0.986, 95% CI = 0.981–0.991] and was highly correlated with the 100MTT walk/run velocity (Spearman rho correlation coefficient of r_s_(134) = .92).

**Conclusion:**

Although LGMD subtypes may differ in age of onset, rate of progression, and patterns of muscle weakness, the overall impact of progressive muscle weakness on motor function is similar. The NSAD is a reliable and valid ClinRO of motor performance for individuals with LGMD and is suitable for use in clinical practice and research settings.

**Impact:**

Recent developments in potential pharmacological treatments for LGMD have highlighted the urgent need for disease-specific outcome measures. Validated and meaningful outcome measures are necessary to capture disease presentation, to inform expected rates of progression, and as endpoints for measuring the response to interventions in clinical trials. The NSAD, a scale of motor performance for both individuals who have LGMD and are ambulatory and those who are nonambulatory, is suitable for use in clinical and research settings.

## Introduction

Limb-girdle muscular dystrophies (LGMD) are a group of progressive, rare, genetically and clinically heterogeneous neuromuscular conditions. All forms of LGMD are characterized by muscle weakness involving the pelvic and/or shoulder girdle muscles.^1^^,^^2^ There is a paucity of natural history studies using standardized methods to characterize these diseases. Clinically meaningful and validated clinician-reported outcome measures (ClinROs) are essential to accurately evaluate disease progression,^3^ to characterize the natural history, and for use as a primary or secondary outcome measure to evaluate the response to novel therapeutic interventions.^4^ The lack of validated ClinROs of motor performance for individuals with LGMD represents a major unmet need, as gene therapy trials and other pharmacological interventions are now within reach for this population.^5^^,^^6^

The prevalence of LGMD worldwide has been estimated at 1.63 per 100,000 population^7^^,^^8^ and at 2.27 per 100,000 in northeastern England,^9^ with variations between countries possibly due to the effects of founder mutations. The autosomal recessive forms of LGMD are more common than the autosomal dominant forms, although both are rare. With rapid progress in gene and protein discovery, over 30 types of LGMD have been identified. The original naming convention for LGMD, identifying each diagnosis with a letter, reached its limit at recessive type Z, and a review of the nomenclature was required. The new naming system replaces the 1 or 2 for inheritance pattern with recessive (R) and dominant (D) and replaces the letter with a number assigned according to the order of discovery of the affected protein.^10^

The development of ClinROs for motor performance requires clinically meaningful and scientifically robust data. Psychometrics, the science of rating scales^4^—specifically, psychometric analysis using a Rasch unidimensional measurement model (RMM)—has been applied in the development and validation of scales of motor performance for neuromuscular disorders; these include the North Star Ambulatory Assessment for Duchenne muscular dystrophy and Performance of Upper Limb 2.0 assessment (PUL).^3^^,^^11^ RMM examines the fit between a clinician’s scoring of an individual’s performance on an item and predictions of those scores from the Rasch model (which defines how a set of items should perform to generate reliable and valid measurements).^12^ RMM estimates an individual’s ability and the item difficulty on a single linear rule on the basis of the responses provided for each item and makes the assumption that individuals who are more able have a greater probability of scoring high on an item than those who are less able. Every individual’s ability and the difficulty of each item are represented by a score, expressed in logits (log odds units), a linear unit defined as the natural logarithm of the odds of positive achievement of any item by an individual. A scale may be considered valid when there is good agreement between the observed and expected scores from the model.^12^^,^^13^ RMM has the ability to confirm if it is appropriate for a scale’s ordinal-level items to be summed to create a total score.^14^

The North Star Assessment for limb-girdle type muscular dystrophies (NSAD) was the first ClinRO of motor performance, developed initially for LGMD R2/2B (dysferlinopathy).^15^ This RMM-built scale was developed from natural history data collected as part of the international multisite clinical outcomes study for dysferlinopathy (NCT01676077).^16^ This scale of motor performance includes items suitable for the measurement of both individuals who are ambulatory and individuals who are not ambulatory. The robust psychometric properties of the NSAD warrant investigation of this scale’s utility in other LGMD subtypes for which disease-specific ClinROs are not available.^15^ Accurate characterization of LGMD subtypes is critical for understanding expected rates of decline and key for the interpretation of future interventional trial results. The purposes of this study were to evaluate the ability of this scale to quantify motor function across the broad spectrum of abilities found in individual cohorts with LGMD, examine the relationship of the NSAD with timed function tests and PUL, and assess the intra- and interrater reliabilities of the NSAD.

## Methods

### Participants

A total of 130 individuals, pediatric and adult, with a confirmed clinical or genetic diagnosis of LGMD participated across 2 sites—Nationwide Children’s Hospital (NCH), Columbus, Ohio (USA), and the John Walton Muscular Dystrophy Research Centre (JWMDRC), Newcastle upon Tyne, UK.

### Standard Protocol Approval, Registration, and Participant Consent

Data were collected across 2 sites, NCH and JWMDRC, by their respective physical therapy teams between June 2016 and March 2021. Ethics approval was obtained from the Institutional Review Board at NCH (IRB 17–01086 and IRB 09–00139) and audit (10055) registered with the Newcastle upon Tyne Hospitals National Health Service Foundation Trust. All individuals recruited under the Neuromuscular Natural History Study at NCH consented and assented, if appropriate, to participate in the trial. Data from the NCH team were obtained during research visits, including the LGMD International Patient Meeting in Chicago, IL (USA), in August 2019. Clinical data were collated by the team at JWMDRC.

### Data Collection

Individual-reported sex and age at assessment and diagnosis were recorded. Consenting individuals completed the functional assessments described further below. The NSAD was completed by all individuals included in the analysis. Data from the 100-m timed test (100MTT) and PUL were collected by the NCH team only because of clinical space limitations at JWMDRC. Teams at these 2 neuromuscular expert centers regularly establish interrater reliability within and across centers at least biennially. Evaluations were carried out with a standardized physical therapy manual and worksheets. In addition, to quantify the reliability of the NSAD, all physical therapists scored video-recorded NSAD assessments and repeated the scoring between 2 and 4 weeks later.

### Assessments

#### North Star Assessment for Limb-Girdle Type Muscular Dystrophies

The NSAD is a 29-item scale of motor performance for both individuals who are ambulatory and are not ambulatory (Suppl. Fig. 1). It includes items such as the ability to roll, sit to stand, jump, rise from the floor (timed), and run/walk for 10 m (timed).^15^ In general, items are scored on a 0- to 2-point scale (4 items are scored from 0 to 1 point) for a maximum score of 54 points, with higher scores indicative of higher abilities. The NSAD is used during routine clinical assessments of individuals with LGMD at JWMDRC and NCH.

#### 100-Meter Timed Test

The 100MTT is a test of maximal ambulatory capacity that quantifies the time required for an individual to traverse 2 full laps around a 25-m-long course as quickly as possible, including running if able to do so safely.^17^ The total time to complete the test is recorded in seconds and can be transformed into velocity by dividing 100 m by the total time to complete the assessment.

#### Performance of Upper Limb 2.0 Assessment

LGMD causes progressive muscle weakness of the pelvic and shoulder girdles. Upper limb function should be screened using a validated clinical outcome assessment, such as the entry item of the PUL. When individuals become nonambulatory or upper limb weakness occurs, closer monitoring of upper limb function is recommended. The PUL is a 22-item scale used to assess upper limb function^11^; it was originally designed for use in Duchenne muscular dystrophy but is now used in LGMD. The PUL evaluates motor performance at the shoulder, elbow, and wrist-hand levels and includes bimanual tasks.^18^ As in the NSAD, items are generally scored on a 0- to 2-point scale (2 items are scored from 0 to 1 point) for a maximum score of 42 points, with higher scores indicative of higher abilities.

### Psychometric Evaluation and Statistical Analysis

Psychometric evaluation completed with the RMM was used to examine NSAD performance in 7 areas: targeting, response categories, fit, reliability, dependency, stability, and unidimensionality.^12^ Available data from individuals with between 1 and 6 assessments were included in this analysis and entered into RUMM 2030 software.^19^

#### Targeting

Scale-to-sample targeting investigates the match between the range of motor function measured by the items of the NSAD and the range of motor function ability of the study population.^4^^,^^20^

#### Response Categories

Item scoring, for example, 0/1/2 in the NSAD, must reflect an ordered continuum of better function or, conversely, disease progression. Scale validity is supported when items demonstrate ordered scoring thresholds. Rasch analysis examines these data statistically and graphically by threshold locations and plots.^21^

#### Fit

The items of a scale must work together (fit) both clinically and statistically; otherwise, a total score is inappropriate. All items should lie within a fit residual SD range of ±2.5 to be considered to represent an appropriate fit to the measured construct. Item fit and person fit to the model are examined with *z* scores. A mean close to 0 and a SD of 1 are expected if person and item fit are good. The third fit statistic is the item-trait interaction statistic, given as a chi-square value; a significant chi-square value indicates misfit.

#### Reliability: Person Separation Index (PSI)

The reliability of the scale was quantified using the PSI^22^ (similar to the Cronbach alpha),^23^ which compares the true scale variance to observed variance. A higher PSI indicates greater reliability.

#### Dependency

The response to 1 item on a scale should not unduly influence the response to another item.^4^^,^^20^ If such influence occurs, measurement estimates can be biased and reliability (PSI) may be artificially elevated. Rasch analysis determines the number of pairs of dependent items.

#### Stability: Differential Item Functioning (DIF)

DIF measures the stability of item performance across subgroups, for example, sex. We examined the NSAD for DIF across sex and age; age was divided into 3 categories: <5 years (to reflect attainment of motor milestones), 5 to 18 years, and adults.

#### Unidimensionality

The items of the scale should measure a single construct, which in this case was functional motor performance. This requirement is fundamental for the Rasch model. Unidimensionality was evaluated using principal components analysis of the residuals and reported as a *t* test value.

### Data Analysis

Data analysis was performed with SPSS software version 26 (IBM SPSS, Chicago, IL, USA). Descriptive statistics were used to quantify participant demographics. The inter- and intrarater reliabilities of NSAD scores were assessed using ICC.^24^^,^^25^ Spearman correlation coefficients^26^ were used to explore the relationships of the NSAD, 100MTT velocity, and PUL.

### Role of the Funding Source

The funder played no role in the design, conduct, or reporting of this study.

## Results

### Study Population

A total of 186 assessments from 130 individuals with 14 different diagnoses were included for analysis (Tab. 1). There were 69 female individuals and 61 male individuals between 2.9 and 64.5 years old, with a mean age of 25.9 years. Forty-nine individuals were <18 years old (38%). Both individuals who were ambulatory and nonambulatory were included; 22 of 130 (17%) were not ambulatory.

**Table 1:** Participant Diagnoses and Assessment Numbers by LGMD Subtype*^a^*

LGMD Subtype		No. of Patients (No. of Assessments Completed)		
Old Name/New Name	Protein	NSAD	100MTT	PUL
1B/	Emery-Dreifuss muscular dystrophy	2 (2)	1 (1)	1 (1)
1C/	Rippling muscle disease	1 (1)	1 (1)	1 (1)
1D/D1	*DNAJB6* related	5 (5)	4 (4)	5 (5)
2A/R1	Calpain 3 related	56 (74)	43 (60)	45 (61)
2B/R2	Dysferlin related	15 (17)	6 (6)	8 (8)
2C/R5	γ-Sarcoglycan related	4 (6)	3 (5)	3 (5)
2D/R3	α-Sarcoglycan related	3 (3)	3 (3)	3 (3)
2E/R4	ß-Sarcoglycan related	15 (49)	15 (43)	15 (41)
2I/R9	Dystroglycan/*FKRP* related	1 (1)	0	0
2 J/R10	Titin related	6 (6)	2 (2)	2 (2)
2 L/R12	Anoctamin 5*/ANO5* related	10 (10)	5 (5)	6 (6)
2S/R18	*TRAPPC11* related	3 (3)	3 (3)	1 (1)
/R23	Laminin α_2_/*LAMA2* related	1 (1)	0	0
Clinical diagnosis	Pending genetic confirmation	8 (8)	3 (3)	4 (4)
Totals		130 (186)	89 (136)	94 (138)

^a^ 100MTT = 100-m timed test; LGMD = limb-girdle muscular dystrophies; NSAD = North Star Assessment for limb-girdle type muscular dystrophies; PUL = Performance of Upper Limb 2.0 Assessment.

### Psychometric Evaluation

Available data from 186 assessments were entered into RUMM 2030 software.^19^ The summary of our findings is shown in Table 2. The NSAD had high reliability, with a PSI of 0.96, and the scale was confirmed to be unidimensional.

**Table 2:** NSAD Psychometric Evaluation Summary Using Rasch Mathematical Methods*^a^*

Parameter	Data
Item fit, mean (SD)	−0.63 (2.01)
Person fit, mean (SD)	−0.28 (0.93)
Reliability (PSI with extremes)	0.96
Ordered thresholds	27/29 (97%)
No. of items with good fit*^b^*	23/29
Dependency (no. of pairs)	9
Unidimensionality	Acceptable (*t* test = 0.037; 95% CI = 0.006–0.069)

^a^ NSAD = North Star Assessment for limb-girdle type muscular dystrophies; PSI = Person Separation Index.

^b^ Defined as a fit residual inside the recommended range (−2.50 to +2.50). Three of the 6 misfitting items had significant χ^2^ probability (*P* < .01).

#### Targeting

**Figure 1:**
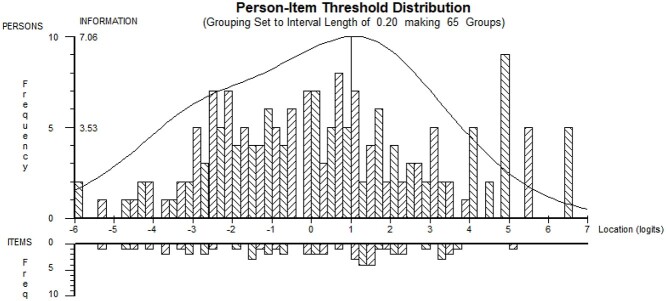
North Star Assessment for limb-girdle type muscular dystrophies (NSAD) person-item threshold distribution plot demonstrating good coverage of items for the population. The top histogram illustrates the abilities of the population, from the weakest on the left to the strongest on the right. The bottom histogram demonstrates a well-distributed range of items that test the ability of the population, with a ceiling for the individuals who were the very strongest and asymptomatic.

Person-item threshold distribution maps were examined for any significant floor or ceiling effects or gaps in the continuum of measurement of the NSAD. Items of the NSAD successfully targeted both cohorts (ambulatory and nonambulatory) (Fig. 1). Item locations were spread from 2.6 to −4.1, indicating a good continuum of coverage with little overlap. A ceiling existed for individuals who were the most strong and asymptomatic, and a floor existed for individuals who were the weakest, with no independent pelvic or shoulder girdle movement. The overall item-trait interaction chi-square value was 494.15 (*df* = 58).

#### Response Categories

**Figure 2:**
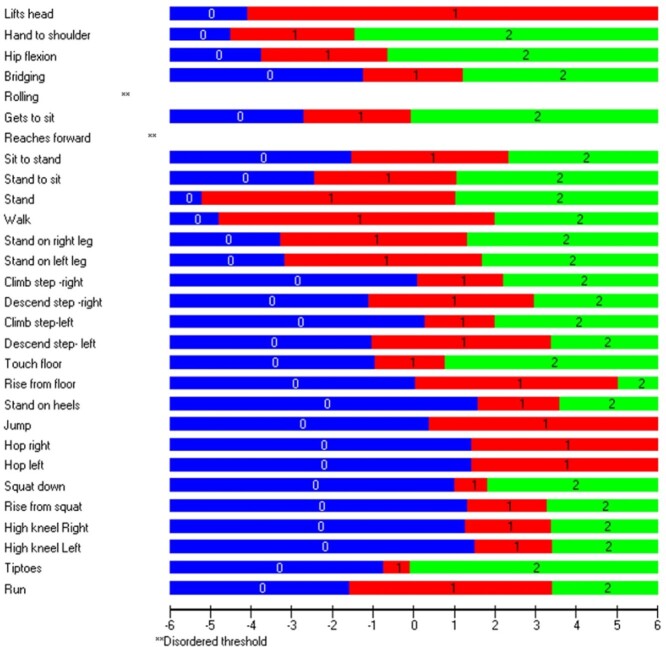
Item thresholds with 27/29 ordered scoring thresholds demonstrating that as an individual’s ability increases, so does the probability of obtaining a higher score.

Twenty-seven of the 29 items presented with ordered response categories (Fig. 2), with the remaining 2 (roll and reach forward) approaching an ordered threshold (Suppl. Fig. 2A and 2B); these results indicated that the scoring of these items followed expected progression and function, as designed.

#### Fit

Six items were misfit, 3 with a chi-square probability <.01 (Tab. 3). The misfit items were sit to stand, stand on 1 leg (right and left), climb box step (right), rise from floor, and stand on tiptoes. In the context of other parameters, these 6 items functioned well, had strong clinical meaning, and were retained in the scale.

**Table 3:** Individual Item Fit for the 29 Items in Threshold Location: Order of Difficulty, From Easiest to Most Difficult

Item	Item No.	Item Location	SE	Fit Residual	χ^2^	χ^2^ *P*
Lift head	I0001	−4.084	0.336	0.56	47.425	0
Roll supine to prone	I0005	−3.386	0.204	0.104	7.44	.024
Hand to opposite shoulder	I0002	−2.979	0.188	0.466	9.988	.007
Reach forward	I0007	−2.265	0.158	1.53	81.728	0
Hip flexion in supine	I0003	−2.181	0.174	−0.024	0.4	.819
Stand	I0010	−2.087	0.197	2.006	2.912	.233
Walk	I0011	−1.385	0.204	−0.971	2.083	.353
Get to sitting	I0006	−1.373	0.16	−1.251	4.46	.107
Stand on 1 leg: right	I0012	−0.974	0.173	3.131*^a^*	17.898	.000
Stand on 1 leg: left	I0013	−0.749	0.175	3.113*^a^*	28.962	.000
Stand to sit	I0009	−0.69	0.163	−0.937	4.522	.104
Stand on tiptoes	I0028	−0.421	0.147	4.846*^a^*	171.891	0
Touch floor from standing	I0018	−0.09	0.152	−1.772	8.76	.012
Bridge in supine	I0004	−0.009	0.155	0.49	0.166	.920
Jump	I0021	0.385	0.213	−2.343	9.723	.008
Sit to stand	I0008	0.408	0.163	−2.987*^a^*	6.19	.045
Walk/run 10 m	I0029	0.919	0.172	−2.426	4.307	.116
Descend box step: right	I0015	0.941	0.166	−2.37	3.74	.154
Climb box step: left	I0016	1.146	0.157	−2.064	5.724	.057
Climb box step: right	I0014	1.165	0.158	−2.723*^a^*	7.011	.030
Descend box step: left	I0017	1.181	0.169	−1.77	4.692	.096
Hop: left	I0023	1.424	0.225	−1.739	8.359	.0153
Squat down	I0024	1.426	0.158	−1.564	2.578	.275
Hop: right	I0022	1.439	0.225	−2.088	11.005	.004
Rise from squat	I0025	2.305	0.173	−1.51	3.965	.138
High kneel to stand: right	I0026	2.328	0.174	−1.81	10.528	.005
High kneel to stand: left	I0027	2.466	0.177	−1.85	11.293	.003
Rise from floor	I0019	2.545	0.181	−2.827*^a^*	11.046	.004
Stand on heels	I0020	2.594	0.178	0.702	5.357	.069

^a^ Item that “misfit” the overall scale (such items should lie in the range of ±2.5).

#### Dependency

Nine pairs of items had highly correlated residuals (>0.4). All pairs were items assessing bilateral performance or component parts of the same movement (ie, squat down and rise from squat). We further examined the PSI with 1 item from each pair of dependent items removed. The PSI remained stable at 0.96, indicating that the dependency did not artificially inflate the PSI and that these items were not causing bias. Inclusion of these bilateral items was deemed clinically significant because they have the potential to improve sensitivity to change in LGMD subtypes with asymmetrical weakness patterns/presentation.

#### Stability (DIF)

The NSAD was examined for the presence of uniform DIF and nonuniform DIF by sex and age. No DIF was identified for sex on any item (sex did not affect the way in which each item was scored). In the under-5-year group, uniform DIF was present on hop (right and left), stand on 1 leg (right and left), and jump. Lift head also closely approached a uniform DIF for age in individuals <5 years. These items are all developmentally attained milestones, supporting the clinical meaning of the presence of DIF on these items.

### Inter- and Intrarater Reliabilities

Interrater reliability was excellent (ICC = 0.986; *P* < .001; 95% CI = 0.981–0.991). Intrarater reliability was also excellent, with the ICCs of individual raters ranging from 0.952 to 1 (*P* < .001; 95% CI = 0.921–1).

### Correlation of the NSAD With 100MTT and PUL

**Figure 3:**
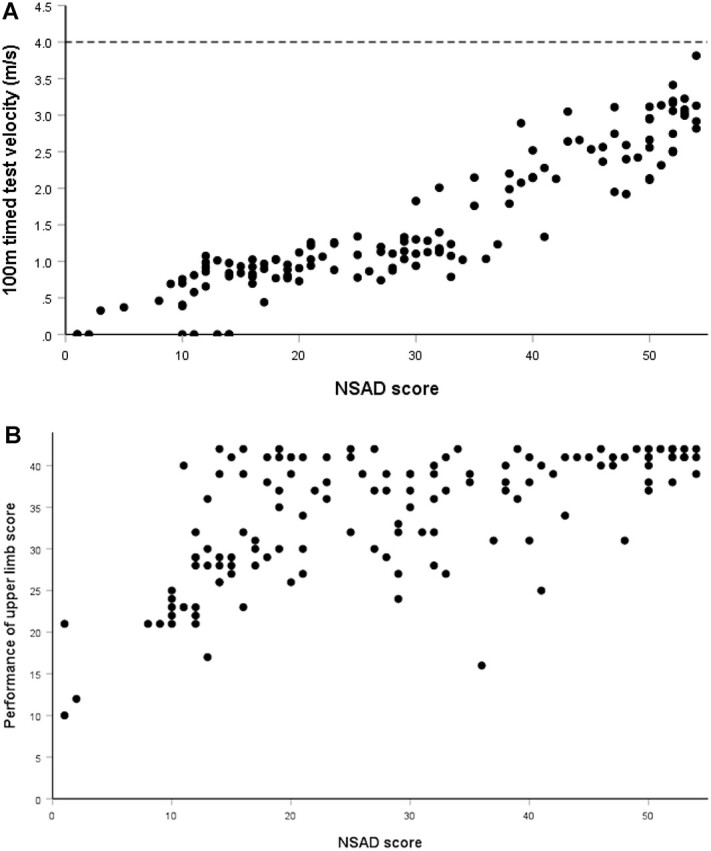
Test correlations. (A) North Star Assessment for limb-girdle type muscular dystrophies (NSAD) total score and 100-m timed test velocity (m/s) scatterplot. There was a strong correlation between motor performance, as measured with the NSAD total score, and run/walk velocity. The line at 4 m/s indicates normal 100-m velocity for children 8 to 14 years old. (B) NSAD total score and Performance of Upper Limb 2.0 assessment score scatterplot. Despite excellent gross motor performance, as indicated by high NSAD scores, some individuals who were ambulatory experienced significant upper limb dysfunction.

100MTT walk/run velocity was highly correlated with the NSAD total score (Fig. 3A) (*r*_s(134)_ = .92; *P* < .001). The inclusion of the 100MTT reduced any potential ceiling effect, because no individual in our current cohort was observed to run at above the reported average speed of 4 m/s in children between 8 and 14 years old.^26^ The NSAD was mildly correlated with the PUL score (*r*_s(136)_ = .65 *P* < .001). Figure 3B illustrates that the PUL discriminated upper limb ability in the cohort of individuals who were weaker on their NSAD score and quantified upper limb dysfunction present in some individuals who were ambulatory and had excellent gross motor ability, with high scores on the NSAD.^27^

## Discussion

In this report, RMM techniques were used to examine the suitability of the NSAD scale as a ClinRO of motor performance in a variety of LGMD subtypes. Although LGMD subtypes may differ in age of onset, rate of progression, and patterns of muscle weakness or involvement, the overall impacts of progressive muscle weakness on motor function are similar. We developed and validated the NSAD, a scale of motor performance for individuals with LGMD, both ambulatory and nonambulatory.

RMM psychometric analysis confirmed that the observed NSAD data showed good fit with the model’s predictions for these ratings. RMM demonstrated that the NSAD provided a unidimensional scale for the measurement of motor performance, and disease progression was reflected in individual item scores. It was reliable and valid for assessing individuals across LGMD subgroups. Items of the scale fit well and had logical scoring to ensure a meaningful total score.

The broad range of items included in the NSAD for individuals with LGMD spanned the spectrum of functional ability from ambulant to nonambulant. The NSAD tests both proximal and distal and anterior and posterior muscles of the lower limbs, with trunk and proximal shoulder items also included.

The presence of significant misfit of the “stand on tiptoes” item was expected, because it is a function that mainly individuals with LGMD R2/2B and LGMD R12/2 L—in which early and significant calf muscle weakness is present—struggle to complete. Of the other 5 misfit items, 2 were related to standing on 1 leg and may be attributed to the variable proximal muscle weakness of the hip. The other 3 only misfit slightly. In children <5 years, the presence of DIF demonstrated a developmental impact on motor skills, such as jumping and hopping.

A subgroup of individuals was found to have a ceiling effect. Further analysis indicated that these individuals were diagnosed through positive family history or after accidental findings in their creatine kinase levels without previous reported symptoms. The strong relationship between NSAD performance and 100MTT performance indicated that the addition of the 100MTT could capture change in a minimally affected cohort of individuals with LGMD.

The relationship between arm function via the PUL and the NSAD illustrated the importance of examining all individuals with LGMD for upper limb involvement. Although upper limb function was generally more affected as gross motor performance deteriorated and individuals became nonambulatory, there were individuals who were strong and ambulatory but had upper limb impairment in this cohort. The PUL entry item could be used as an upper limb screening tool in all individuals with LGMD.

### Limitations

The NSAD was initially developed and validated for use in LGMD 2B or R2 dysferlin-related cohort, and the results from our study confirmed the utility of the scale in a variety of LGMD subtypes. A concern was whether the distribution of subtypes in the study cohort, a sample of convenience, mirrored the prevalence of subtypes in the general population of individuals with LGMD. The currently published prevalence of subtypes varies greatly within and between countries. At our 2 highly specialized neuromuscular centers, across 2 continents, we see individuals from a wide geographical spread across the United Kingdom and United States and often internationally too. We used an international conference of LGMD patients with attendees from around the world. There was good representation in our cohort with 14 different LGMD subtypes, and the largest number of patients had LGMD R1, the most prevalent LGMD.

A limitation of the present study was that we used only cross-sectional analysis. Longitudinal data collection is under way to determine the responsiveness of the scale across LGMD subtypes, although the scale has demonstrated excellent sensitivity over 1 year in the LGMD R2/2B population.^15^ Ongoing work will explore the suitability of the scale for other neuromuscular diseases that present with limb-girdle weakness. In addition, examination of the relationship of the NSAD to patient-reported outcome measures is being undertaken.

ClinROs of motor performance are used to determine clinical trial outcomes,^6^^,^^28^ construct accurate natural history data,^15^^,^^17^ and make clinical patient care decisions.^4^^,^^29^ The appropriateness of these outcomes, based on the use of a ClinRO, is dependent on the scientific qualities of a scale. Our psychometric analysis using RMM supports the NSAD as a robust scale for LGMD. The present study illustrated the value of Rasch analysis, which adds sophistication and refinement to traditional psychometric methods and provides detailed diagnostic item-level data. Applying the Rasch model in conjunction with physical therapist clinical knowledge of the disease and outcome measures allows for the refinement of each item while ensuring clinical utility.

Recent developments in treatments for LGMD subtypes have highlighted the urgent need for disease-specific outcome assessments. To our knowledge, the NSAD is the first psychometrically built ClinRO of motor performance specifically designed for LGMD and should be considered for both clinical and research applications.

## Supplementary Material

PTJ-2021-0943_R1_Supplementary_Figure_1_pzac113_ddac186

PTJ-2021-0943_R1_Supplementary_Figure_2_pzac113

## Clinical Trial Registration

The NSAD was developed from natural history data collected as part of the international multisite Clinical Outcomes Study for dysferlinopathy (NCT01676077).
