# Supplementary material for: Validation of the North Star Assessment for Limb-Girdle Type Muscular Dystrophies
Source: Phys Ther. 2022 Aug 6;102(10):pzac113. doi: 10.1093/ptj/pzac113 (PMC9586158; doi:10.1093/ptj/pzac113)
Supplement: PTJ-2021-0943_R1_Supplementary_Figure_1_pzac113_ddac186 [file ptj-2021-0943_r1_supplementary_figure_1_pzac113_ddac186.zip › PTJ-2021-0943_R1_Supplementary_Figure_1_pzac113_ddac186.pdf]

Supplementary Figure 1. NSAD worksheet Version 1.1 Copyright 2018 The Newcastle upon Tyne Hospitals NHS Foundation Trust. This worksheet and accompanying manual available from authors or via opentact.net.

Centre ID____Pt ID_________Pt Initials_____Date _ _ / _ _ _ / _ _ _ _Assessor____________Signature____________

North Star Assessment for Limb Girdle Type Muscular Dystrophies (NSAD) worksheet				
Activity\Score	2	1	0	Achieved Score
1. Lifts head in supine		In supine, head must be lifted in mid-line. Chin moves towards chest	Unable. No clearance of head from surface or only partially achieved movement, uses protraction or side flexion	
2. Hand to opposite shoulder	Raises hands and moves it to opposite shoulder, no compensation	Raises hand and moves it to opposite shoulder using compensatory movements	Unable. Does not achieve finish position	R L
3. Hip flexion in supine	In supine, flexes hip and knee more than 90° by raising foot off the bed through whole movement	Partially flexes hip and knee (<90°, > 20°) or foot remains in contact with mat. Or uses external rotation of the hip	Unable. Hitches hip only or raises less that 20° of hip flexion	R L
4. Bridging in supine	Maintains start position for count of 5 then full bridge count of 5, kneecaps pointing up, feet slightly apart, knees not touching	Maintains the starting position for 5 seconds. May or may not partially raises the pelvis off mat	Unable to maintain start position for count of 5	
5. Rolling supine to prone	Turns over into prone and frees elbows from under trunk	Rolls partially / at least onto side (vertical pelvis and shoulder girdle)	Unable or only by pulling on edge of bed	
6. Gets to sitting	Able to sit up using one hand / arm to push up	Uses two arms / pulls on legs, turns towards floor or uses momentum /rocking	Unable. Or has to put legs over the side of the bed in order to sit up	
7. Reaches forwards	Without upper limb support, leans forward, 30° or more and sits back again	With upper limb support, leans forward 30° or more and sits back again	Unable or flexes trunk <30°	
8. Stand up from chair	Able to stand up from a start position of 90° hip and knee flexion, keeping arms folded and feet shoulder width apart.	Pushes on thighs or chair using one or two hands/ prone turn or altering start position by widening base	Unable or uses external support	
9. Stand to sit on chair	Able to sit down without using arm support in a controlled way	With arm support, able to sit down in chair safely	Unable to sit down without assistance/ uncontrolled	
10. Stand	Stands upright and symmetrically, without compensation (with heels flat and feet facing forward in neutral) for minimum count of 3 seconds	Stands but with some degree of compensation	Cannot stand independently, needs support	
11. Walk	Walks consistently with heel-toe or flat-footed gait pattern. "Normal" gait pattern.	Adapted walking pattern e.g. wide base, altered foot posture, waddling, foot drop or other gait deviation	Loss of independent ambulation – may use orthosis or walk short distances with assistance	
12. Stand on one leg - right	Able to stand in a relaxed manner (no fixation) for count of 3 seconds	Stands <3 seconds, or momentarily or with trunk side-flexion >20° or needs fixation e.g. by thighs adducted	Unable / needs external support	
13. Stand on one leg - left	Able to stand in a relaxed manner (no fixation) for count of 3 seconds	Stands <3 seconds, or momentarily or with trunk side-flexion >20° or needs fixation e.g. by thighs adducted	Unable / needs external support	
14. Climb box step - right	Faces step / no compensation / no support needed	Goes up sideways / rotates trunk / circumducts hip/ widens base of support or needs hand for balance or with any other compensation	Unable or if weight bears through evaluators hand	

 (NSAD) © 2018 The Newcastle upon Tyne Hospitals NHS Foundation Trust

Centre ID____Pt ID_________Pt Initials_____Date _ _ / _ _ _ / _ _ _ _Assessor____________Signature____________

Activity\Score	2	1	0	Achieved Score
15. Descend box step -right	Faces forward, steps down controlling weight bearing leg / No support needed	Goes down sideways / rotates trunk / circumducts hip/ widens base of support or needs hand for balance or with any other compensation	Unable or if weight bears through evaluators hand	
16. Climb box step - left	Faces step / no compensation / no support needed	Goes up sideways / rotates trunk / circumducts hip/ widens base of support or needs hand for balance or with any other compensation	Unable or if weight bears through evaluators hand	
17. Descend box step -left	Faces forward, steps down controlling weight bearing leg / No support needed	Goes down sideways / rotates trunk / circumducts hip/ widens base of support or needs hand for balance or with any other compensation	Unable or if weight bears through evaluators hand	
18. Touches floor from standing	Without support, touches floor with one hand and stands up again	With support (arm on floor, body, plinth) touches floor with one hand and stands up again	Unable / able to touch floor but cannot get back up again	
19. Rise from floor	Starts in long sitting - No evidence of Gower's manoeuvre.	Gower's manoeuvre- rolls towards floor or uses both hands on the floor and/or use hand(s) on legs	(a) NEEDS to use external support object e.g. chair OR (b) Unable<br>No time recorded	Time (00.0s):<br>__ __ . __s
20. Stands on heels	Both feet at the same time, clearly standing on heels only (acceptable to move a few steps to keep balance) for count of 3	Raises both forefeet (must raise all metatarsal heads off the floor) or dorsiflexes one foot.	Unable or 5th metatarsal remains on floor or foot inversion/supination	
21. Jump		Both feet at the same time, clears the ground simultaneously	Unable	
22. Hop right leg		Entire foot clears the floor. Start and finish on one leg	Unable / Foot not leaving the floor	
23. Hop left leg		Entire foot clears the floor. Start and finish on one leg	Unable / Foot not leaving the floor	
24. Squat down	Squats down fully with arms free (more than 90° of hip and knee flexion)	Uses one or two hands on thighs or floor to assist full squat – can tick both<br>On thigh<br>On floor	Unable / Partial bend at hip and knee	
25. Rise from squat	Stands up from full squat without using arms / hands on floor or legs	Uses one or two hands to rise from squat to stand – can tick both<br>On thigh<br>On floor	Unable to get back up from squat without assistance/uses furniture/change out of squat position	
26. High kneel to stand through R leg lead with L leg	Able with arms free	Able to stand up through R half kneeling using hands on floor or legs (Furniture NOT allowed)	Unable / needs external support	
27. High kneel to stand through L leg lead with R leg	Able with arms free	Able to stand up through L half kneeling using hands on floor or legs. (Furniture NOT allowed)	Unable / needs external support	
28. Tiptoes	Tiptoes on one foot, clearly on toes for 3 seconds, knee straight	Tiptoes on both feet at the same time, clearly on toes for 3 seconds, knees extended	Unable / momentarily / only with knee flexed (not using active plantar flexion)	
29. Run (10 m)	Runs- both feet off the ground, achieves flight (both feet off floor simultaneously)	Fast walk or jog or ability to increase their normal walking speed	Walks, with no increase in speed possible<br>OR<br>Unable to walk 10 m	Time (00.0s):<br>__ __ . __s

RFF time _ _._ sec 10m run / walk time _ _._ sec Total score _ _ / 54

(NSAD) © 2018 The Newcastle upon Tyne Hospitals NHS Foundation Trust
