# Supplementary material for: Validation of the North Star Assessment for Limb-Girdle Type Muscular Dystrophies
Source: Phys Ther. 2022 Aug 6;102(10):pzac113. doi: 10.1093/ptj/pzac113 (PMC9586158; doi:10.1093/ptj/pzac113)

Supplementary Figure 2. Category probability curves for (A) rolling and (B) reaches forward items. The placement of score 1 curve (red) near to the intersection of score 0 and 2 suggests it is close to reaching an ordered threshold in both items.

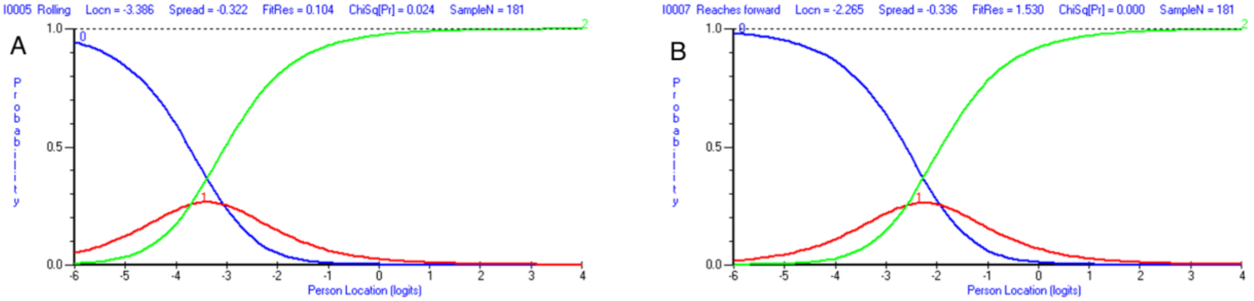

Supplement: PTJ-2021-0943_R1_Supplementary_Figure_2_pzac113 [file ptj-2021-0943_r1_supplementary_figure_2_pzac113.pdf]
